# The prevalence and clinical correlates of adverse childhood experiences in a cross-sectional study of primary care patients with cardiometabolic disease or risk factors

**DOI:** 10.1186/s12872-019-01277-3

**Published:** 2019-12-19

**Authors:** Robert G. Maunder, David W. Tannenbaum, Joanne A. Permaul, Melissa Nutik, Cleo Haber, Mira Mitri, Daniela Costantini, Jonathan J. Hunter

**Affiliations:** 1 Department of Psychiatry, Sinai Health System and University of Toronto, Toronto, Canada; 2 Ray D. Wolfe Department of Family Medicine, Sinai Health System, University of Toronto, Toronto, Canada; 3 Department of Family and Community Medicine, University of Toronto, Toronto, Canada; 4 Mount Sinai Academic Team, Ray D. Wolfe Department of Family Medicine, Sinai Health System, University of Toronto, Toronto, Canada

**Keywords:** Childhood adversity, Cardiovascular risk, Prevention, Quality of life

## Abstract

**Background:**

Adverse childhood experiences (ACEs) are associated with risk of poor adult health, including cardiometabolic diseases. Little is known about the correlates of ACEs for adults who have already developed cardiometabolic diseases, or who are at elevated risk.

**Methods:**

Adult primary care patients with cardiometabolic disease (hypertension, diabetes, stroke, angina, myocardial infarction, coronary artery bypass graft, angioplasty) or with a risk factor (obesity, smoking, high cholesterol, family history) were surveyed regarding ACEs, psychological distress, attachment insecurity, quality of life, behavior change goals, stages of change, and attitudes toward potential prevention strategies.

**Results:**

Of 387 eligible patients, 74% completed the ACEs survey. Exposure to ACEs was reported by 174 participants (61%). Controlling for age, gender, relationship status and income, number of ACEs was associated with psychological distress (F = 3.7, *p* = .01), quality of life (F = 8.9, *p* = .001), attachment anxiety (F = 3.4, *p* = .02), drinking alcohol most days (F = 4.0, *p* = .008) and smoking (F = 2.7, *p* = .04). Greater ACE exposure was associated with less likelihood of selecting diet or physical activity as a behavior change goal (linear-by-linear association *p* = .009). Stage of change was not associated with ACEs. ACEs exposure was not related to preferred resources for behavior change.

**Conclusions:**

ACEs are common among patients at cardiometabolic risk and are related to quality of life, psychological factors that influence cardiometabolic outcomes and behavior change goals. ACEs should be taken into account when managing cardiometabolic risk in family medicine.

## Background

Adverse childhood experiences (ACEs), which include exposures to neglect, abuse and family dysfunction, are common and are associated with increased risk of poor health outcomes in adulthood. Using a definition of ACEs that includes material neglect and abuse, physical abuse, sexual abuse, emotional neglect and abuse, parental separation, and exposure to family violence, drug use, or mental illness, Felitti found a graded relationship between the number of childhood ACE exposures and the prevalence of a range of adult diseases that are major contributors to early mortality [1]. Subsequently, these associations have been replicated in a large, representative population sample drawn from several U.S. states [2] and extended to many health risk behaviors [3] and poor health outcomes [4], including early mortality [5]. Exposure to at least one ACE is consistently reported by 50–60% of adults, with smaller numbers reporting multiple exposures [1, 2].

There is consistent evidence from childhood through later adulthood that ACEs are both associated with cardiometabolic risk and with plausible behavioral and physiological mechanisms that cause cardiovascular disease. In childhood ACEs are correlated with changes in heart rate, body mass index, and waist circumference [6]. In adolescents and young adults, ACEs are associated with greater peripheral resistance, arterial stiffness, increased blood pressure, and elevated circulating endothelin-1 levels [7]. Systolic and diastolic blood pressure increase more quickly after age 30 in adults who have been exposed to ACEs than in those who have not [8]. For adults, childhood adversity is also associated with lifestyle risk factors, including physical inactivity, smoking, and obesity [1, 9–11]. Excess cardiometabolic risk, after accounting for lifestyle factors, may be the result of associations of ACEs to major depression, high inflammation levels, high blood pressure, high total cholesterol, low high-density lipoprotein cholesterol, high glycated hemoglobin, and low maximum oxygen consumption levels [12]. Presumably as a result of these behavioral and physiological consequences of ACEs, exposure to ACEs, especially exposure to more than 2 or 3 types of ACEs, is associated with ischemic heart disease, stroke and type 2 diabetes [1, 2, 13–15], although not all studies are consistent [4]. In January, 2018, the American Heart Association published a position statement which concluded that “substantial evidence links childhood adversity to cardiometabolic disease later in the life course, including heart disease, type 2 diabetes mellitus, and stroke” [16].

Much less is known about the correlates of ACEs for adults who have already developed cardiometabolic disease, or who are at elevated risk of it based on conventional risk factors, such as family history, hypertension, or smoking. Although in general exposure to ACEs is associated with psychosocial factors that add risk and/or burden to physical illness such as depression [12, 17, 18], attachment anxiety, attachment avoidance [19–21] and lower health-related quality of life (QOL) [22], it is not known how or if these variables are related to ACEs in patients who either have cardiometabolic disease or who are at elevated risk for it. Supporting lifestyle changes is a central component of the management of cardiac risk in primary care. Although ACEs may be related to symptoms or attitudes that affect health behavior, it is not known if patients who have cardiometabolic disease or who are at elevated risk of it and have exposure to ACEs have different preferences for interventions that provide support or promote lifestyle changes.

In this study, we hypothesized that among primary care patients with cardiometabolic disease or at elevated risk of cardiometabolic disease, ACEs would be correlated with high psychological distress, low quality of life, attachment insecurity, smoking and hazardous drinking (as this exposure is in the general population). Since preferences for interventions that support behavior change and mental health had not previously been studied, we explored the relationship of ACEs with these variables without a hypothesis.

## Methods

This is a cross-sectional study of an at-risk cohort of primary care patients, which includes both patients who have cardiometabolic disease and patients with at least one conventional risk factor. Adult patients at two academic family health team sites in Toronto, Canada were approached in the waiting room, between August 2016 and January 2017, by a research assistant who described the study and obtained verbal consent from interested patients. Those who consented were screened for cardiometabolic risk factors to determine eligibility.

Eligible patients were 18 years of age and older, English-speaking, and independently able to complete the survey. They had to report at least one of the following: body mass index > 30; current smoker; high blood pressure; high cholesterol; diabetes; history of stroke, angina, myocardial infarction, coronary artery bypass graft (CABG), or angioplasty prior to the past 3 months; or history of a first degree relative with stroke, angina, myocardial infarction, CABG, or angioplasty before the age of 60. Patients with a history of stroke, angina, myocardial infarction, CABG, or angioplasty in the past 3 months were excluded because the recency of acute illness might influence psychological characteristics and preferences.

Eligible patients were asked to provide written consent and then complete the study survey either electronically or on paper. Patients who were unable to complete the survey in the waiting room were given the option to complete it at home. Participants also consented to collection of information from their electronic medical record (EMR). Information on objective cardiovascular risk (BMI, blood pressure, LDL, TC/HDL ratio, Hba1c, diagnosis of hypertension, diabetes, previous CV event, and medications) was gathered from the EMR to confirm screening self-report, to get data for Framingham risk calculations and to provide data for relevant secondary analyses. When there was a discrepancy between information from the patient and from the EMR, it was resolved by using the source we considered most likely to be valid (e.g. patient report for smoking status, EMR for high cholesterol). Information about treatment with specific medication classes was used to complete the Framingham score of patients’ risk status. A sample size of 300 was targeted to provide a confidence interval of ±5.5 percentage points on an expected prevalence of ACEs of 50%.

### Survey Design & Instruments

The survey collected demographic data including socioeconomic status, and measured quality of life, psychological distress, alcohol use, stages of change with respect to diet, exercise, or smoking, attachment insecurity and attitudes toward an array of potential prevention strategies.

QOL was measured with the Quality of Life Enjoyment and Satisfaction Questionnaire – Short Form [23], a 16-item self-report measure, designed to assess the degree of enjoyment and satisfaction experienced in various areas of daily functioning on a 5-point scale from very poor (1) to very good (5). Results are reported as percentage of maximal score, providing an easily interpreted scale from 0 to 100.

Self-rated health was measured with a visual analogue scale from the EuroQol Quality of Life Scale [24], on which a participant indicates their health on a 100-point scale from “worst imaginable health state” (0) to “best imaginable health state” (100).

Attachment insecurity was measured with the modified 16-item Experience in Close Relationships (ECR M-16) [25], which has been validated in older and medically ill patients [26]. Attachment anxiety and attachment avoidance are each measured on a 7-point scale from 1 to 7, with higher scores indicating greater attachment insecurity. For interpretation of scores, it is noted that in adults who completed the ECR-M16 in an online resource [26], mean scores and standard deviations for people with or without self-reported significant health problems were as follows. Medically ill (*N* = 240): attachment anxiety 4.0 ± 1.5, attachment avoidance 3.4 ± 1.3; Not medically ill (*N* = 227): attachment anxiety 3.8 ± 1.3, attachment avoidance 3.1 ± 1.3.

Psychological distress was measured with the Kessler Psychological Distress Scale (K10) [27], a scale which uses 5 items probing the frequency of anxiety symptoms and 5 items probing depressive symptoms, each on a scale from “none of the time” (0) to “all of the time” (4), yielding a total score from 0 to 40. For interpretation of scores, prior testing as a screening instrument indicated a score > 12 was maximally sensitive and specific in predicting any mood or anxiety disorder as defined in DSM-IV [28, 29].

Potential alcohol use disorder was measured with the Alcohol Use Disorders Identification Test (AUDIT-C) [30], a 3-question alcohol screen that can help identify patients who are hazardous drinkers or have active alcohol use disorders (including alcohol use or dependence). Three multiple choice questions probe frequency of alcohol consumption in a month, typical number of drinks in a sitting, and frequency of drinking 6 or more drinks in a sitting in a month.

The Adverse Childhood Experiences (ACE) questionnaire was used to probe 10 types of adversity that may have occurred before age 18 (emotional abuse, emotional neglect, physical abuse, physical neglect, sexual abuse, parental separation, violence against mother, household member with mental illness, addiction or incarceration) [1]. Each type of adversity is scored as either present or absent, yielding an ACE score between 0 and 10.

The study team also developed the Helping You Reach Your Goals questionnaire to assess patients’ behavior change plans and which behavior change interventions they would consider. This measure surveyed preferred targets of behavior change (change in diet, increased physical activity, smoking cessation or other), preferences for the goals of potential resources (from a list of 31 resources that involve others in behavior change, provide encouragement, track behavior change, treat mental illness, help with feelings, or provide information) and types of resources (individual, group, or independent self-directed resources).

Stage of change with respect to the preferred behavior change (pre-contemplative, contemplative, preparation, action, or maintenance) was measured with the question assessing stage of change with respect to exercise in the Weight Loss Behavior Stage Change Scale [31], modified to address the participant’s preferred behavior change target.

### Analysis

Descriptive statistics were used to characterize the cohort. Exposure to adversity was categorized as endorsement of zero, one, two or three, or four or more ACEs. Bivariate relationships between behavioral or psychological variables and demographic variables were tested by Kruskal-Wallis test, Mann-Whitney U test, Spearman rank-order correlation or Chi^2^ test as appropriate. Relationships between ACE category and psychological/behavioral dependent variables (psychological distress, QOL, self-reported health, attachment anxiety, attachment avoidance, BMI, smoking status, six or more drinks in a sitting at least monthly, typically more than 2 drinks in a sitting, drink alcohol more than three times a week), controlling for age, gender, relationship status and income, were tested by multivariate analysis of variance (MANOVA). Levene’s test of equality of error variances was significant for the alcohol and smoking variables in the MANOVA and so these were removed and the MANOVA was repeated without them. The relationship of ACE category to alcohol and smoking variables was subsequently tested in a series of univariate analyses of variance (ANOVAs).

In order to explore the relationship between ACE exposure and intentions with respect to behavior change, we first tested the association between ACE exposure category and the preferred target of behavior change by Chi^2^ test. We also tested the relationship between ACE exposure and stage of change with respect to the preferred target, controlling for age, gender, relationship status and income, by univariate ANOVA. Finally, we used ANOVA to explore if specific types of ACE exposure are differentially associated with health outcomes, selecting QOL as an exemplar of outcome because of its strong relationship with ACE category (multiplicity of ACE exposures).

## Results

Of 996 patients approached to participate in the study; 604 (61%) agreed to be screened; 387 (64%) were eligible for the study; and 309 (80% of eligible patients) completed the survey. Of those who completed the survey, 286 (74% of those eligible) who completed the ACEs component of the survey form the cohort for the current analysis. Characteristics of the participants are provided in Table 1. Noteworthy characteristics are that the sample was approximately equally divided between men and women, the median age was 59 years (range 18–92, inter-quartile range 46–69), the modal annual income category was between $90,000 and $120,000 (CAN), and most participants (58%) were married or in common-law relationships. Half of participants (50%) were included because of cardiometabolic disease or cardiovascular events (hypertension, diabetes, stroke, myocardial infarction, angina, history of coronary artery bypass surgery, cardiac stent or angioplasty), and the other half were included because of cardiovascular risk factors (smoking, high cholesterol, family history of cardiovascular disease before age 60) in the absence of disease.

**Table 1:** Characteristics of participants

	*N*	%	Mean	SD
Gender				
Male	126	44.1		
Female	159	55.5		
Other	1	0.3		
Annual Income ($1000 CAN)				
29,999 or less	33	11.5		
30,000 – 59,999	27	9.4		
60,000 – 89,999	21	7.3		
90,000 – 119,999	60	21.0		
120,000 – 149,999	47	16.4		
150,000 or more	35	12.2		
Didn’t say/prefer not to answer	63	22.0		
Relationship Status				
Single	41	14.3		
Married or common-law	167	58.4		
Separated, divorced or widowed	60	21.0		
Other/prefer not to answer	18	6.3		
Inclusion criteria met (self-report)				
Smoker	59	17.5		
Hypertension	123	43.0		
High cholesterol	98	34.3		
Diabetes	35	12.2		
Stroke	16	5.2		
MI	13	4.5		
Angina	16	5.6		
CABG	8	2.8		
Angioplasty/stent	17	5.9		
Family history	138	48.3		
Framingham Risk Category				
Data not available to calculate	71	24.8		
Low risk	129	45.1		
Intermediate risk	45	15.7		
High risk	41	14.3		
Body Mass Index				
Up to 25	90	31.5		
> 25 to 30	108	37.8		
> 30 to 40	70	24.5		
> 40	16	5.6		
Frequency of drinking alcohol				
Never	49	17.1		
Monthly	67	23.4		
2–4 times a month	73	25.5		
2–3 times a week	42	14.7		
4 or more times a week	47	16.4		
Typical number of drinks				
None, I don’t drink	57	19.9		
1 or 2	161	56.3		
3 or 4	43	15.0		
5 or 6	10	3.5		
7 to 9	4	1.4		
10 or more	2	0.7		
Frequency of drinking 6 or more				
Never	202	70.6		
Less than monthly	46	16.1		
Monthly	18	6.3		
Weekly	8	2.8		
Daily or almost daily	6	1.7		
Preferred Behaviour Change				
Healthier diet	108	37.8		
Increase physical activity	115	40.2		
Stop smoking	19	6.6		
Other	34	11.9		
Stage of Change				
Pre-contemplative	22	7.7		
Contemplative	59	20.6		
Preparation	52	18.2		
Action	70	24.5		
Maintenance	59	20.6		
Exposure to adverse childhood experiences (ACE score)				
None	112	39.2		
One	66	23.1		
Two	43	13.9		
Three	13	4.2		
Four	12	3.9		
Five	17	5.5		
Six	13	4.2		
Seven	4	1.3		
Eight	5	1.6		
Nine	0	0		
Ten	1	0.3		
Self-rated health (0 to 100)			73.0	16.6
Quality of Life and Satisfaction (% of maximum score)			70.5	16.9
Psychological Distress (0 to 40)			7.7	6.9
Attachment anxiety (1 to 7)			3.0	1.3
Attachment avoidance (1 to 7)			3.0	1.2

Exposure to ACEs was reported by 174 participants (61%). The number of ACEs reported by participants is provided in Table 1. For analysis, ACEs were categorized as none (39%), one (23%), two or three (20%), or four or more (18%). ACE exposure was significantly greater in participants with lower income and who were not in married or common-law relationships (data not shown). These demographic characteristics were included in subsequent analyses as covariates.

Age was also included as a covariate in subsequent analyses because it was associated with several relevant variables, detailed in Table 2. In particular, age was associated with the reason for inclusion in the study, with older participants more likely to have experienced cardiometabolic disease or cardiovascular events. Younger age was associated with a higher prevalence of current or recent smoking, and two indicators of high alcohol use: drinking six or more drinks in a sitting at least monthly, and typically drinking 3 or more drinks in a sitting. In addition, younger age was associated with higher cumulative exposure to ACEs. Although cumulative ACE exposure was greater in younger cohorts, most specific ACE exposures did not differ between age groups. Only separation or divorce, having a family member incarcerated, and emotional abuse were more prevalent in lower age groups. Note that the significance of the latter does not survive the Bonferroni correction for ten comparisons (i.e. when significance is set at *p* < .005).

**Table 2:** Relationship between age and other variables

	Age group (years)				Difference between groups	
	18–40 *N* = 49	41–50 *N* = 43	51–60 *N* = 61	> 60 *N* = 133	Chi^2^	*p*
Cardiovascular disease vs. risk factors only						
History of cardiometabolic disease or cardiovascular events^a^	20.4%	32.6%	47.5%	67.7%	39.1	<.001
Substance Use						
Current smoker or quit in last year	40.8%	25.6%	16.4%	6.8%	31.1	<.001
Alcohol at least 4 days per week^b^	10.4%	16.3%	15.3%	20.3%	2.6	.54
Six drinks or more per sitting at least monthly^c^	25.5%	16.3%	11.7%	3.9%	17.9	<.001
Typically, 3 drinks or more per sitting^d^	48.9%	25.6%	22.4%	9.3%	33.0	<.001
Cumulative exposure to ACEs						
No ACEs	28.6%	48.8%	29.5%	44.4%		
One ACE	18.4%	11.6%	24.6%	27.8%		
Two or three ACEs	24.5%	18.6%	24.6%	15.8%		
Four or more ACEs	28.6%	20.9%	21.3%	12.0%	17.6	.04
Exposure to specific ACEs						
Physical abuse	18.8%	16.3%	19.7%	12.8%	1.9	.59
Sexual abuse	10.4%	16.3%	18.0%	12.8%	1.7	.64
Emotional abuse	36.7%	30.2%	37.7%	18.8%	10.5	.02
Emotional neglect	29.2%	16.3%	24.6%	17.6%	3.9	.27
Material deprivation	10.4%	9.3%	9.8%	6.8%	0.9	.82
Parental separation	47.9%	16.7%	21.3%	15.9%	21.9	<.001
Family violence	12.8%	14.6%	8.3%	8.4%	1.9	.59
Family substance use	20.8%	23.8%	27.9%	16.5%	3.5	.32
Family mental illness	35.4%	26.2%	27.9%	17.3%	7.3	.06
Incarceration of family member	16.7%	4.8%	4.9%	0.8%	16.1^e^	<.001

^a^Hypertension, diabetes, stroke, myocardial infarction, angina, history of coronary artery bypass surgery, cardiac stent or angioplasty

^b^Missing data from 8 subjects

^c^Missing data from 7 subjects

^d^Missing data from 9 subjects

^e^Fisher’s Exact test

In MANOVA, controlling for age, gender, relationship status and income, ACE category was significantly associated with the dependent variables (Wilk’s Lambda = 0.82, *p* = .01). Between-subjects effects showed a significant relationship between ACE category and psychological distress (F = 3.7, p = .01), QOL (F = 8.9, *p* = .001), and attachment anxiety (F = 3.4, *p* = .02) and no significant relationship with attachment avoidance (F = 1.6, *p* = .19), self-rated health (F = 1.5, *p* = .22) and BMI (F = 1.55, *p* = .20). Univariate ANOVAs, controlling for age, gender, relationship status and income, showed that ACE category had a significant relationship with drinking alcohol on 4 or more days a week (F = 4.0, *p* = .008) and smoking (F = 2.7, *p* = .04) and no significant relationship with drinking 6 or more drinks in one sitting at least monthly (F = 1.1, *p* = .33), or typically drinking 3 or more drinks in a sitting (F = 2.3, *p* = .08).

**Fig. 1:**
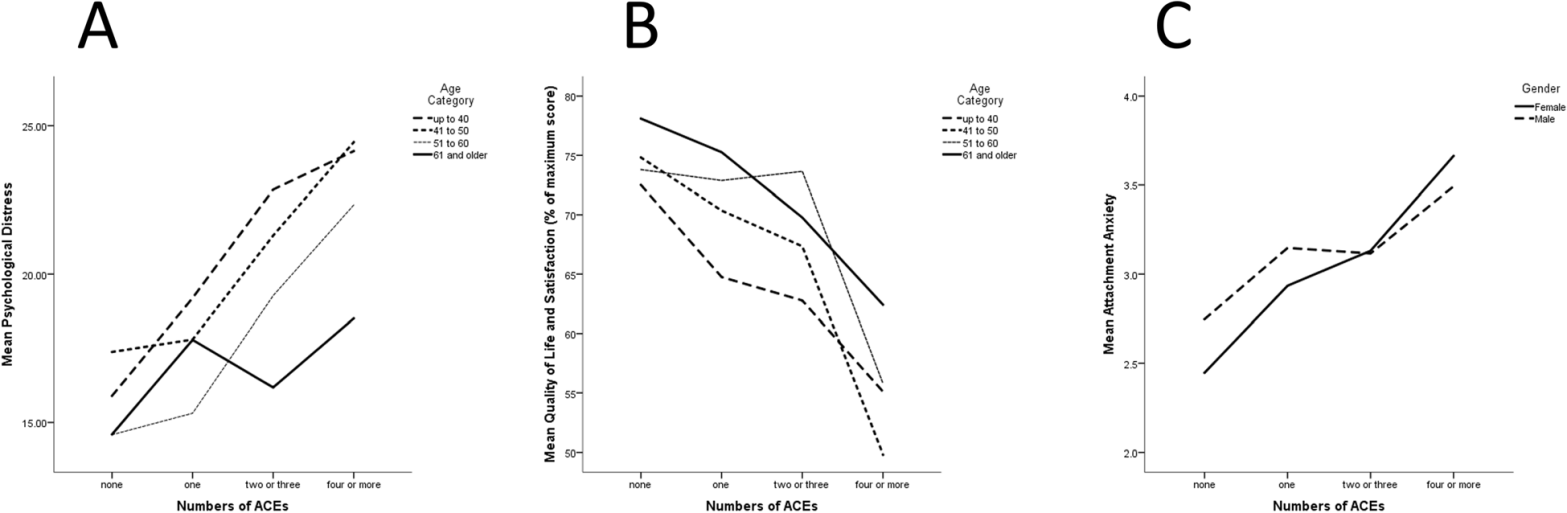
Relationship between ACE exposure and (**a**) psychological distress, (**b**) quality of life and (**c**) attachment anxiety

The direction and pattern of the significant relationships between ACE and three clinical variables are shown in Fig. 1. Results in these figures are divided by age and/or gender when these demographic variables were significant in analysis of variance. There was a strong linear relationship between ACE exposure and psychological distress in all age cohorts except the oldest group (Fig. 1a). Figure 1b illustrates a strong relationship between greater exposure to ACEs and lower quality of life that is consistent in different age cohorts. Figure 1c shows attachment anxiety increases with increasing ACE exposure in both men and women.

Regarding health behaviors that were related to ACE (as demonstrated above), current smoking was more common with greater exposure to ACEs among both men and women and was more common in participants who were single, separated or divorced. The relationship between ACE category and drinking alcohol on 4 or more days a week was non-linear, with the peak prevalence of this frequent alcohol consumption in people exposed to 2 or 3 ACEs with lower prevalence at lower and higher exposures to ACEs.

**Fig. 2:**
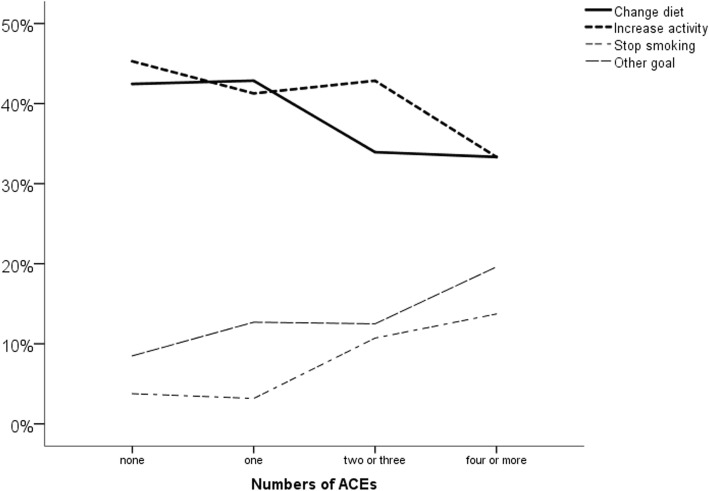
Relationship between ACE category and patients’ goals for behavior change

Regarding behavior change, ACE exposure was significantly related to the goal of behavior change that participants selected (linear-by-linear association *p* = .009). As shown in Fig. 2, increasing ACE exposure was associated with less likelihood of selecting a change in diet or physical activity as a goal and a greater likelihood of choosing smoking cessation or selecting another goal. Among participants with two or more ACEs, the items written in as “other” goals were: decrease alcohol (2), improve sleep (2), be able to afford to go to gym (1), reduce anxiety (1), be better organized (1), all of the above (1), and “not making changes” (1). Stage of change with respect to the selected behavior change was not associated with ACE exposure (F = 0.2, *p* = .90) after controlling for age (F = 0.1, *p* = .77), gender (F = 0.0, *p* = .99), relationship status (F = 4.5, *p* = .04) and income (F = 0.5, *p* = .46). As shown in Table 3, ACE exposure was not related to the resources for behavior change that participants would consider: neither to the goals of those resources (gathering information, coping with interfering feelings, tracking progress, receiving reminders and encouragement, treating mental illness or engaging others in support), nor to the mode of support (group resources, individual resources or resources that can be used independently). Post-hoc testing of the direction of relationship between age and these resource preferences indicated that in each case where age was related to preferences, older age was associated with lower likelihood of preference for resources, regardless of specific goals or intervention or type of resource (data not shown).

**Table 3:** Relationship between ACE category and preferences for resources for behavior change

Dependent Variable	Covariates								Independent Variable	
	Age		Gender		Relationship Status		Income		ACE Category	
	F	Sig.	F	Sig.	F	Sig.	F	Sig.	F	Sig.
Goal of resources										
Involve others	6.0	.02	0.0	.91	3.0	.09	3.9	.05	2.1	.11
Provide encouragement	3.9	.05	2.8	.09	0.5	.49	0.8	.38	0.5	.70
Track behavior change	11.1	.001	0.3	.62	0.2	.69	0.4	.51	0.9	.43
Treat mental illness	6.8	.01	0.2	.64	0.1	.73	1.3	.25	1.7	.17
Help with feelings	10.9	.001	0.2	.67	0.1	.78	1.4	.23	0.6	.62
Provide information	2.4	.12	0.2	.67	0.9	.36	0.9	.35	1.0	.40
Type of resources										
Individual resources	11.7	.001	0.5	.50	1.2	.27	3.1	.08	1.5	.21
Group resources	1.2	.27	0.4	.53	0.1	.73	8.8	.003	0.1	.94
Independent resources	11.8	.001	1.2	.27	2.5	.12	.23	.64	0.1	.97

In order to address the question of the relative impact of particular types of ACE exposure, we compared the effect of having or not having exposure to each of the ten categories of ACE to quality of life. As shown in Table 4, the effect was significant for each type of ACE and the size of the effect was similar as well, with exposure to ACE accounting for between 2 and 11% of the variance (eta^2^) in quality of life.

**Table 4:** The association of individual types of ACE exposure to quality of life

Type of ACE	Prevalence	Exposure present		Exposure absent				
		Quality of Life^a^		Quality of Life				
		Mean	SD	Mean	SD	F	Sig	Effect size (eta^2^)
Emotional abuse	27.8%	61.7	17.9	73.8	15.3	32.7	<.001	.10
Physical abuse	15.8%	59.2	18.4	72.6	15.8	25.9	<.001	.08
Sexual abuse	14.1%	64.8	18.3	71.4	16.6	5.3	.02	.02
Emotional neglect	20.8%	59.6	18.0	73.3	15.6	33.9	<.001	.11
Material deprivation	8.4%	58.4	21.9	71.6	16.0	14.0	<.001	.05
Separation or divorce	22.5%	63.4	20.8	72.6	15.2	15.2	<.001	.05
Family violence	9.9%	58.4	19.4	71.7	16.2	16.2	<.001	.06
Family drug use	20.8%	62.2	18.8	72.7	15.8	19.0	<.001	.06
Family mental illness	23.9%	62.0	19.3	73.2	15.2	24.5	<.001	.08
Incarceration of family member	4.9%	48.5	20.2	71.7	16.0	27.2	<.001	.09

^a^Quality of life reported from 0 to 100 as the percent of maximum score of a scale from 1 (very poor) to 5 (very good)

## Discussion

Individuals who have been exposed to ACEs are at greater risk of developing cardiometabolic disease [16]. To our knowledge, this is the first study to investigate the incidence and clinical correlates of ACEs in primary care patients who already have cardiometabolic disease or are at elevated risk based on conventional risk factors. The prevalence of ACEs in the current study was similar to what we have previously found in adult primary care patients in our setting who were unselected for cardiometabolic risk [11]. Results of the current study show that for patients with cardiometabolic disease or at elevated risk, a history of ACE is relevant for its relationship to QOL, psychological factors that influence CVD outcome (psychological distress, attachment insecurity) as well as health behavior (smoking, hazardous drinking) and behavior change goals.

In this study, ACE exposure was associated with lower QOL. Corso and colleagues also found that adults who reported maltreatment in childhood had significantly reduced health-related QOL compared to those who did not experience maltreatment [22]. Our finding that exposure to ACEs is associated with greater psychological distress (depressive and anxiety symptoms) and with attachment anxiety also corroborates previous studies in people who are not selected for cardiometabolic disease or risk [12, 17, 19], which have found a strong association between childhood adversity and depressive symptoms, antisocial behavior, drug use, suicidal ideation, and insecure attachment style. In these respects, adults with and without cardiovascular disease and risk factors appear similar.

Contrary to previous studies of the general adult population [1, 10, 11], ACE exposure was not significantly associated with body mass index or glycemic control in this study. This may be an indication that the determinants of risk behavior are different in people at known cardiac risk compared to the general population. When considering a behavior change goal, we found that ACE exposure did not predict the types of interventions that individuals favoured (e.g. group vs. individual counselling, or independent use of resources that could assist with goal attainment). Although most patients chose dietary change or increased physical activity as their primary behavior change goal, smoking cessation was chosen more often in those with higher levels of ACE exposure (beyond that expected by their increased rate of smoking).

The relationship between age and several of the variables (and the relationships between variables) assessed in this study is clinically relevant. As expected, cardiovascular and cardiometabolic disease is more common among older patients. As also expected, smoking and indicators of problematic alcohol use are more common among younger patients. While cumulative ACE exposure is significantly greater among younger patients, it cannot be determined from the current data if this is a cohort effect (changing patterns of children exposed to adversity over time) or a reporting effect (decreasing likelihood to report adversity with advancing age). A similar trend has been reported in other studies [32]. It is noteworthy in this respect that most specific types of adversity were reported at similar rates at all ages. Thus, there is little evidence of changes in the types of adversity experienced by children over time (with the exception of increasing parental separation and an increase in exposure perceived as emotionally abusive in younger people). Importantly, the relationships found between ACEs and smoking, drinking 4 or more days a week, psychological distress, quality of life, and attachment anxiety are significant after controlling for effects of age. Indeed, the slopes of the relationships between ACEs and either psychological distress or QOL are quite similar in most age cohorts (Fig. 1a and b). With respect to preferences for behavior-change resources, it appears that younger patients may be more likely to wish to engage in resources, especially individual and independent (self-help) resources that target involving others in their care, providing encouragement, tracking behavior change, treating mental illness and addressing difficult emotions. Given that younger patients are also more likely to have conventional risk factors that have not yet progressed to cardiovascular and cardiometabolic disease, this suggests an opportunity for prevention.

This study has some limitations. Incidents of childhood maltreatment were self-reported, with the possibility that recollection was limited by recall bias or that incidents were intentionally under-reported. The cross-sectional design of the survey prevents drawing causal conclusions about the associations that were found. Furthermore, we cannot assess if patients’ preferences about behavior change goals and resources are linked to actual behavior.

The findings of this study suggest that there may be benefit in primary care to utilizing a trauma-informed care model among patients who have, or are at risk for, cardiometabolic disease and who disclose a background of ACEs, in order to attempt to reduce the risk for primary or secondary cardiovascular events through preventive interventions. This care model, which focuses on relationship building, acquiring an understanding of the patient’s past experiences, assuming a patient-centred and non-judgmental approach, emphasizing patients’ strengths, avoiding re-traumatization and demonstrating patience with the pace of change [33], may be of particular importance toward reducing future health risks in these patients. Importantly, previous surveys suggest that it is uncommon in primary care even to ask about ACE [34, 35], which suggests that introducing discussion of childhood adversity into primary care “in the same way that [family physicians] ask about other risk factors for health” [36] may be an important first step towards primary and secondary prevention. Opportunities to engage patients in preventive behavior change may be greatest in younger patients.

## Conclusions

In primary care patients who have cardiometabolic disease or are at elevated risk based on conventional risk factors, a history of ACE is associated with lower QOL, health behavior, behavior change goals and with psychological factors that influence CVD outcome, such as psychological distress and attachment anxiety. ACE exposure did not predict the types of behavior change interventions that individuals favoured. Cumulative ACE exposure is significantly greater among younger patients, but it is not known if this is a cohort effect or a reporting effect. The findings of this study suggest that there may be benefit in primary care to utilizing a trauma-informed care model among patients who have, or are at risk for, cardiometabolic disease and who disclose a background of ACEs, in order to attempt to reduce the risk for primary or secondary cardiovascular events.

## Data Availability

The datasets analyzed during the current study are available from the corresponding author on reasonable request.

## Abbreviations

ACEs: Adverse childhood events
ANOVA: Analysis of variance
AUDIT: Alcohol use disorders identification test
BMI: Body mass index
CABG: Coronary artery bypass graft
CV: Cardiovascular
DSM-IV: Diagnostic and statistical manual of mental disorders, fourth edition
ECR: Experience in close relationships
EMR: Electronic medical record
Hba1c: Hemoglobin a1c
HDL: High density lipoprotein
K10: Kessler psychological distress scale
LDL: Low density lipoprotein
MANOVA: Multivariate analysis of variance
QOL: Quality of life
SD: Standard deviation
TC: Total cholesterol
